# Edible Mushrooms as Source of Fibrin(ogen)olytic Enzymes: Comparison between Four Cultivated Species

**DOI:** 10.3390/molecules27238145

**Published:** 2022-11-23

**Authors:** Tania Petraglia, Tiziana Latronico, Grazia Maria Liuzzi, Angela Fanigliulo, Aniello Crescenzi, Rocco Rossano

**Affiliations:** 1 Department of Sciences, University of Basilicata, 85100 Potenza, Italy; 2 Department of Biosciences, Biotechnologies and Environment, University of Bari “Aldo Moro”, 70126 Bari, Italy; 3 Bioagritest Srl—Centro Interregionale di Diagnosi Vegetale, 85010 Pignola, Italy; 4 School of Agricultural, Forestry, Food and Environmental Sciences, University of Basilicata, 85100 Potenza, Italy

**Keywords:** cultivation, edible mushrooms, fibrin(ogen)olytic activity, fibrin, fibrinogen, enzymes

## Abstract

Cardiovascular diseases represent the main cause of death. A common feature of cardiovascular disease is thrombosis resulting from intravascular accumulation of fibrin. In the last years, several fibrinolytic enzymes have been discovered in many medicinal or edible mushrooms as potential new antithrombotic agents. This study aimed to compare the fibrin(ogen)olytic activity of crude extracts from the fruiting bodies of four cultivated edible mushrooms: *Lentinula edodes*, *Pleurotus ostreatus*, *Pleurotus eryngii*, and *Agrocybe aegerita*. Fibrin(ogen)olytic activity was assessed by fibrin plate, spectrophotometric assay and electrophoretic analysis (SDS-PAGE and zymography). The highest activity was detected for *P. ostreatus* followed by *P. eryngii*, *L. edodes* and *A. aegerita*. Results indicated that enzymes exhibited maximum activity at pH 6–7 and 30–40 °C, respectively. Enzyme activity was inhibited by serine and metalloprotease inhibitors. We proposed a new index called the Specific Fibrin(ogen)olytic Index (SFI), which allows specification of the proportion of the total proteolytic capacity due to the fibrin(ogen)olytic activity. These data suggest that the extracts from fruiting bodies or powdered mushrooms can be used as functional ingredients for the development of new functional foods that may act as thrombolytic agents responding, at the same time, to the increasing demand for safe, healthy and sustainable food.

## 1. Introduction

Cardiovascular diseases represent the main cause of death [1]. Among the various risk factors for vascular diseases, intravascular thrombosis, resulting from intravascular accumulation of fibrin, undoubtedly represents one of the most important factors. Fibrin is generated from soluble fibrinogen by the action of thrombin, while its degradation occurs by plasmin. Physiologically, the formation of fibrin clots and fibrinolysis are regulated through a form of dynamic balance which guarantees at the same time the coagulability and fluidity of the blood [2]. However, the imbalance caused by some disorders can result in failure in the process of dissolving fibrin clots, thus leading to serious problems such as pulmonary embolism, vascular disease, aortic aneurysm, myocardial infarction and stroke [3]. The antithrombotic agents used in clinical practice belong to two classes of drugs: anticoagulants (such as vitamin K antagonists and heparins) and fibrinolytic agents (such as tissue plasminogen activator (t-PA), streptokinase and urokinase). However, they exhibit similar adverse effects which includes bleeding, hypotension, allergic reactions, angioedema, and reperfusion arrhythmias (when used in acute heart attack). In addition, they have a short half-life, low specificity for fibrin and a high production cost [4,5]. In the last two decades, several studies have been conducted in the search for new antithrombotic agents from natural sources, characterized by high therapeutic efficacy and low side effects, to be used as supplements or as alternatives to the antithrombotic drugs currently in use. In this regard, several fibrinolytic enzymes from various sources such as fermented products, snake venom, marine species, plants, earthworms, microorganisms and insects have been discovered [6,7,8,9,10,11]. In addition, fibrinolytic proteases were also discovered in the fruiting bodies and mycelia of many medicinal or edible mushrooms [12], including *Agrocybe aegerita*, *Ganoderma lucidum*, *Armillaria mellea*, *Tricholoma saponaceum*, *Cordyceps militaris*, *Pleurotus ferulae*, *Pleurotus ostreatus* and *Pleurotus eryngii* [13,14,15,16,17,18,19,20,21]. Mushrooms are edible fungi that possess excellent organoleptic properties, high nutritional value and many biological activities such as immunomodulatory, hepatoprotective, antitumor, anti-inflammatory, antiviral, hypoglycemic, hypolipidemic, hypocholesterolemic, anticancer and antioxidative [22,23,24,25,26,27]. Due to the significant content of proteins, glucans, fibers, unsaturated fatty acids, phenolic compounds, minerals and secondary metabolites, associated with a low lipid content, mushrooms have been appreciated as nutraceuticals and functional ingredients [28,29,30,31]. Mushrooms belong to the phylum Basidiomycota, which includes more than 2000 edible/medicinal species. In recent years the cultivation of mushrooms has undergone a significant increase all over the world and this trend seems set to continue in the years to come [32]. In the present study four cultivated edible mushroom species: *Lentinula edodes*, *Pleurotus ostreatus*, *Pleurotus eryngii* and *Agrocybe aegerita*, have been assessed for their fibrin(ogen)olytic activity. These mushroom species were commercially cultivated, they were highly appreciated both for their organoleptic characteristics in the culinary field and for their healthy properties. This study aimed to compare the fibrin(ogen)olytic activity of the crude extracts obtained from the fruiting bodies of mushrooms by the fibrin plate method, spectrophotometric assay and electrophoretic analysis (SDS-PAGE and zymography).

## 2. Results and Discussion

**Figure 1:**
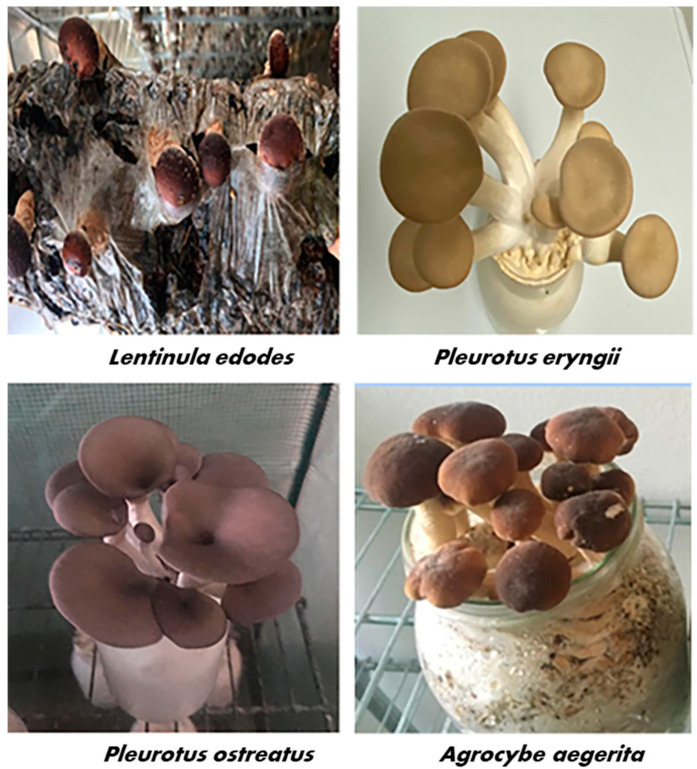
Fruiting bodies of *Lentinula edodes*, *Pleurotus ostreatus*, *Pleurotus eryngii* and *Agrocybe aegerita* cultivated under controlled conditions in the Bioagritest mycological research center (Interregional Center for Plant Diagnosis, Pignola, Italy) and used for the preparation of crude extracts.

The need to have new fibrinolytic enzymes exhibiting low adverse effects, long half-life, high specificity for fibrin and low production cost, as adjuvants or alternatives to traditional drugs, has directed research towards the discovery of new natural sources. In recent years, many fibrinolytic enzymes have been discovered in mushrooms [12]. In the present study, four cultivated edible mushroom species (Figure 1), namely *Lentinula edodes* (Berk.) Pegler (popularly known as shiitake); *Pleurotus ostreatus* (Jacq.) Kummer (known as the oyster mushroom); *Pleurotus eryngii* (DC.) Quélet (commonly known as the king oyster mushroom) and *Agrocybe aegerita* (Brig.) Vizzini (commonly known as Pioppino in Italy), have been assessed for their fibrin(ogen)olytic activity. Although the presence of fibrinolytic enzymes in these mushrooms is already documented in the literature [13,18,20,21], here we compared the fibrin(ogen)olytic activity of crude extracts from the fruiting body of these fungi, considering their use as functional ingredients, based on their nutritional potential and on content of healthy bioactive molecules.

### 2.1. Protein Content and Total Proteolytic Activity Assessment

As shown in Table 1 among the four mushrooms, the highest protein concentration of 9.81 ± 0.39 mg/g of dry weight was observed in crude extracts of *Agrocybe aegerita* (Aae), followed by *Pleurotus eryngii* (Per) (9.02 ± 0.20 mg/g DH) and *Pleurotus ostreatus* (Pos) (8.32 ± 0.15 mg/g DH), while Lentinula edodes (Led) showed the lowest values (7.67 ± 0.25 mg/g DW). By contrast, the highest value of total proteolytic activity was observed for the crude extract from Pos (107.65 ± 6.24 U/mg prot) followed by Per (92.89 ± 3.26 mU/mg prot), whereas the crude extract of Led and Aae showed the lowest activity (85.03 ± 2.32 and 84.69 ± 1.91 mU/mg prot, respectively).

### 2.2. Fibrin Plate Assay

Table 2 reports the fibrinolytic activity of crude mushroom extracts on fibrin plates. All samples showed fibrinolytic activity, represented as lytic area, with diameters ranging from 2.10 to 2.80 cm. Similar to that observed for the total proteolytic activity, the crude extract of *P. ostreatus* was the most active toward fibrin, showing the largest lytic zone with diameter of 2.80 ± 0.13 cm, corresponding to 4.25 ± 0.19 μg of plasmin equivalents (PE), followed by *P. eryngii* displaying a lytic area with diameter of 2.50 ± 0.11 cm (corresponding to 3.41 ± 0.15 μg of PE), whereas in the crude extracts from *L. edodes* and *A. aegerita* we detected the lowest activity, with a lytic diameter of 2.30 ± 0.10 and 2.10 ± 0.07 cm, corresponding to 2.89 ± 0.12 and 2.42 ± 0.13 μg of PE, respectively. The range of values of lytic area observed in this study were similar to that described by Mohamed Ali et al. [33] in extracts from ten different edible mushrooms, including *L. edodes*, but were higher than the lytic zones reported by other authors for *Auricularia polytricha* (1.95 cm) and *Cordyceps militaris* (1.03 cm) [34,35].

### 2.3. Spectrophotometric Detection of the Fibrin(ogen)olytic Activity

**Figure 2:**
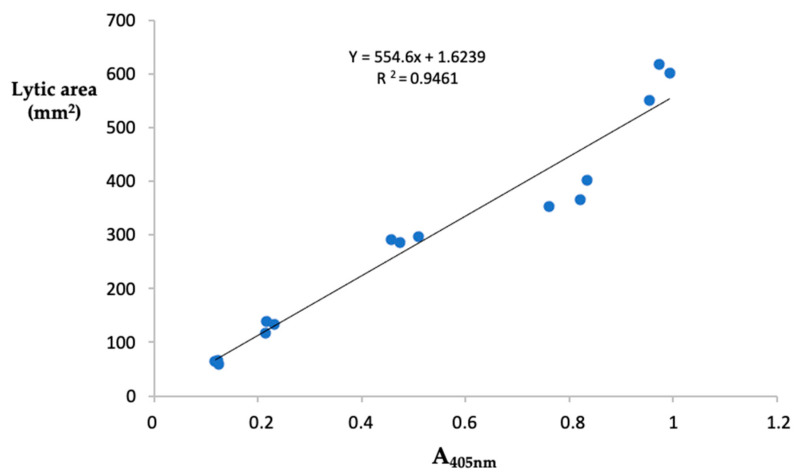
Correlation between fibrin plate assay and spectrophotometric method. Relationship between the absorbance at 405 nm of the released p-nitroanilide from the chromogenic substrates tosyl-Gly-Pro-Lys-p-nitroanilide and the area of lysis on fibrin plate, obtained from the analysis of five different concentrations of plasmin performed in triplicate.

The fibrin(ogen)olytic activity of samples was detected spectrophotometrically using the synthetic substrate tosyl-Gly-Pro-Lys-p-nitroanilide, specific for plasmin. Results, expressed as plasmin equivalents/ mg protein, showed that the highest fibrin(ogen)olytic activity was detected in the crude extracts from *P. ostreatus* (39.14 ± 1.01 µg of PE/mg protein) whereas *A. aegerita* displayed the lowest activity (22.11 ± 0.13 µg of PE/mg prot). The values of the fibrin(ogen)olytic activity found in the extracts from the other samples were: 35.09 ± 0.40 and 23.25 ± 0.16 µg of PE/mg prot for *P. eryngii* and *L. edodes*, respectively (Table 1). These findings are consistent with the results obtained with the fibrin plate assay. In this regard, we evaluated the relationship between the two methods of analysis by measuring the Pearson’s correlation coefficient, considering as variables the absorbance at 405 nm and the area of lysis measured by analyzing five different concentrations of plasmin in triplicates. As reported in Figure 2, the two methods of analysis showed high correlation (r^2^ = 0.946). In this study we proposed a new index called Specific Fibrin(ogen)olytic Index (SFI), corresponding to the ratio between the activity of fibrin(ogen)olytic enzymes and total proteolytic activity. The SFI index, which allows specification of the proportion of the total proteolytic capacity due to the fibrin(ogen)olytic activity showed that the mean value calculated for the two species of *Pleurotus* was 1.4-fold higher than the other two species of mushrooms analyzed.

In previous papers from other authors, the activity of fibrinolytic enzymes was measured spectrophotometrically using various synthetic chromogenic substrates. Cha et al. [20] measured the activity of a fibrinolytic enzyme purified from the fruiting body of *Pleurotus eryngii* using eight different synthetic substrates and found that the fibrinolytic enzyme exhibited the highest activity for the substrate tosyl-Gly-Pro-Lys-p-nitroanilide, the same used in our experiments. Sakovich et al. [36] purified a metalloprotease of 45 kDa in the cultural liquid of *Pleurotus ostreatus*, having high specificity for the chromogenic substrate Leu-pNa. This enzyme showed similarity with the fibrinolytic enzyme described by Choi and Shin [18], purified from the fruiting body of *P. ostreatus*.

#### 2.3.1. Effect of pH and Temperature on the Fibrin(ogen)olytic Activity 

**Figure 3:**
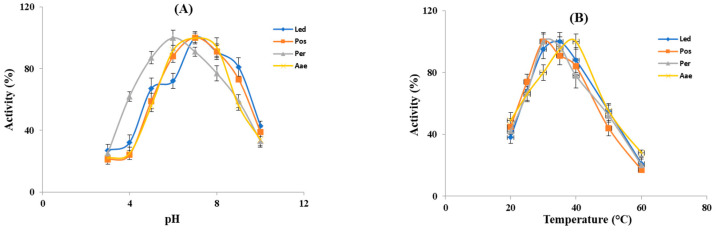
Effect of pH and temperature on the fibrin(ogen)olytic enzymes. (**A**) Optimal pH for the enzyme activity of crude extracts was evaluated within a pH range of 3–10 at 37 °C. (**B**) Optimal temperature for the enzyme activity of crude extracts was determined within a temperature range of 20–60 °C. Led: *L. edodes*, Pos: *P. ostreatus*, Per: *P. eryngii*, Aae: *A. aegerita*.

The influence of pH and temperature on the fibrin(ogen)olytic activity was measured in the crude extracts from the four studied mushrooms. As shown in Figure 3A all the crude extracts displayed fibrin(ogen)olytic activity over a wide pH range (3–10) and exhibited the maximum activity at pH 6–7. In particular, except for *P. eryngii* (Per), which showed the optimal fibrin(ogen)olytic activity at pH 6, all the other samples showed the highest activity at pH 7, which is close to the physiological pH of humans. At pH 3–4, the fibrin(ogen)olytic activity ranged from 20 to 45%, while at pH 9 it was comprised between 50–70%, whereas at pH 10 the fibrin(ogen)olytic activity decreased to 30–40%. As already reported, the optimal pH for mushroom fibrinolytic enzymes is within a pH range of 4–9 [12], although, most of them have optimal pH between 7 and 7.6. In particular, the fibrinolytic enzyme ACase, isolated from the fruiting body of *A. aegerita* exhibited optimal fibrinolytic activity at pH 7.6 [13], whereas the optimal pH of the purified fibrinolytic enzyme from *Pleurotus ostreatus* was 7.4 [37] similar to that found for the proteolytic activity present in our crude extracts from the same mushrooms and for the fibrinolytic enzymes from *Cordyceps militaris* [17] and *Armillaria mellea* [15]. By contrast, as reported by other authors, the purified fibrinolytic enzymes from *Pleurotus eryngii* and *Lentinula edodes* exhibited the maximum activity at pH 5 [20,21], which is different from the optimal pH found in our experiments in the crude extracts of the same mushrooms. This difference is probably due to the presence in the crude extracts from *Pleurotus eryngii* and *Lentinula edodes* of several fibrin(ogen)olytic activities. As reported in Figure 3B the fibrin(ogen)olytic enzymes present in the crude extracts were active within a temperature range of 20–60 °C. In particular, the crude extracts from the genus *Pleurotus* (Per and Pos) exhibited an optimum activity at 30 °C, whereas for *L. edodes* (Led) and *A. aegerita* (Aae) the maximum fibrin(ogen)olytic activity was observed at 35 °C and 40 °C, respectively. At 60 °C, the fibrin(ogen)olytic activity dramatically decreased to 15–25%. As reported by other authors the majority of fibrinolytic enzymes showed an optimal temperature between 35 °C and 50 °C [12]. In other studies, purified fibrinolytic enzymes from *P. eryngii*, *P. ostreatus* and *A. aegerita* exhibited maximum activity at 40, 45 and 47 °C, respectively [13,20,37], higher than the optimal temperature reported for *L. edodes* (30 °C) and *Cordyceps militaris* (37 °C) [17,21].

#### 2.3.2. Effect of Specific Inhibitors

The effect of various inhibitors on fibrin(ogen)olytic enzymes was assessed by measuring residual enzyme activity after incubation of crude extracts with protease inhibitors (Table 3). In the extract from the fruiting body of *L. edodes*, the activity was strongly inhibited (95.5%) by the metalloprotease inhibitor 1.10-Phenantroline (PA) but was not affected by the other inhibitors used. In the crude extracts from *P. eryngii***,** *P. ostreatus* and *A. aegerita*, enzyme activity was inhibited both by the serine protease inhibitor phenylmethyl sulfonyl fluoride (PMSF) and by PA, but not by the aspartic protease inhibitor pepstatin as well as by the cysteine protease inhibitor iodoacetamide (IAA). Based on these findings it can be assumed that our extracts contain different fibrinolytic enzymes which belong to the serine proteases and metalloproteases, as already demonstrated by other authors in several studies [12,13,20,21,37].

### 2.4. Analysis of the Degradation Pattern of Fibrinogen and Fibrin by SDS-PAGE

**Figure 4:**
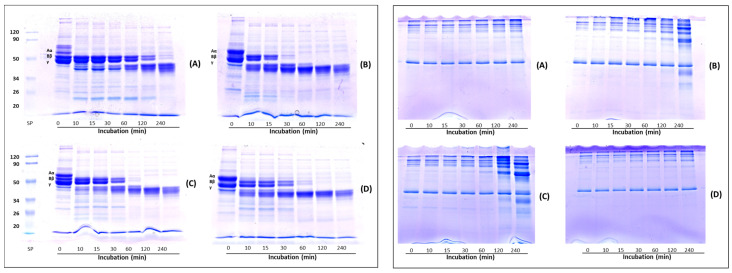
Analysis of fibrin(ogen)olysis patterns by SDS-PAGE. Fibrinogenolysis (**left panel**) and fibrinolysis (**right panel**) patterns exhibited by crude extracts from *Lentinula edodes* (**A**), Pleurotus ostreatus (**B**), *Pleurotus eryngii* (**C**) and *Agrocybe aegerita* (**D**). Crude extracts were incubated with 0.4% human fibrinogen or 0.2% human fibrin in 20 mM Tris-HCl, pH 6.8 at 37 °C, then aliquots of the reaction mixture were removed at different time intervals and analyzed by SDS-PAGE.

Figure 4 represents the fibrinogenolysis (left panel) and fibrinolysis (right panel) pattern exhibited by the crude extracts from *Lentinula edodes* (A), *Pleurotus ostreatus* (B), *Pleurotus eryngii* (C) and *Agrocybe aegerita* (D). Fibrinogen is composed of Aα-, Bβ- and γ-chains, corresponding to the bands indicated on the SDS-PAGE. All samples degraded the Aα-chain preferentially, followed by the Bβ- and γ-chains. The Aα-chain was totally degraded within 10 min, whereas the Bβ- and γ-chain bands in the gels gradually decreased over time. Regarding the Bβ-chain, the four extracts showed different hydrolytic capacities. In gel A, corresponding to the *L. edodes* extract, the Bβ-chain band disappeared after 240 min, in gel B (*P. ostreatus*) after 30 min, whereas *P. eryngii* (gel C) and *A. aegerita* (gel D) completely hydrolyzed the Bβ-chain after 60 min. The γ-chain was degraded after 240 min of incubation in gel A and after 60 min by the other extracts (gel B, C and D, respectively). Concomitant with the digestion of fibrinogen, two major degradation fragments of apparent molecular weight between 45 and 40 kDa were observed. Cha et al. [20] reported on a fibrinolytic enzyme isolated from the fruiting body of *P. eryngii* able to hydrolyze the Aα- Bβ-chains of fibrinogen within 5 and 10 min of incubation, respectively, whereas the complete hydrolysis of γ-chain occurs after 6 h of incubation. The fibrinolytic enzyme ACase, isolated from the fruiting body of *A. aegerita*, degraded all the three chains (α, β and γ) of fibrinogen within 1 min, 10 min and 1.5 h, respectively [13], whereas, the fibrinolytic enzyme purified from the culture supernatant of *P. ostreatus*, hydrolyzed Aα- Bβ-chains within 3 and 45 min, respectively, while the γ chain was degraded slowly over 10 h [37]. The rate of hydrolysis of the fibrinogen subunits, observed in our experiments, which is slower in comparison to the data reported in the literature, could be ascribed to the lower amount of enzyme used. Indeed, other authors evaluated fibrinogen hydrolysis using purified enzymes, which possess higher specific activity than our enzymatic extracts. The right panel shows the analysis of the fibrin degradation pattern. Among the four samples, only the crude extracts from the *Pleurotus* species (gels B and C) were able to degrade fibrin, whereas no hydrolysis was observed with *L. edodes* and *A. aegerita* extracts (gels A and D, respectively) after 240 min. In particular, after 120 min of incubation, the *P. eryngii* extract showed higher fibrinolytic activity than *P. ostreatus*, as evidenced by the presence of more intense degradation bands. However, *L. edodes* and *A. aegerita* began to hydrolyze fibrin after 480 min (data not shown).

### 2.5. Zymographic Analysis of Fibrinolytic and Fibrinogenolytic Activities 

**Figure 5:**
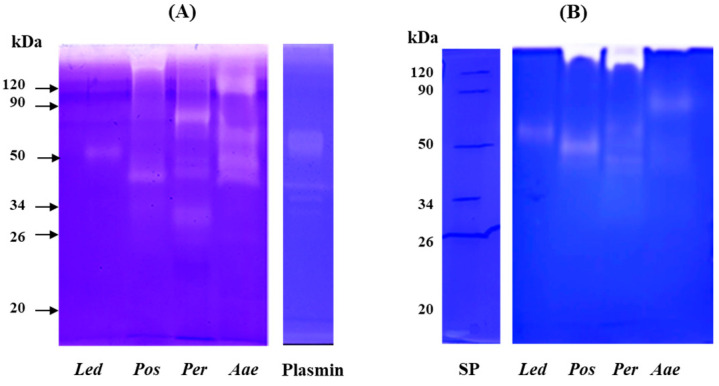
Analysis of fibrinolytic and fibrinogenolytic activity by zymography. Zymograms were carried out under non-reducing conditions on 10% (*w*/*v*) polyacrylamide gels copolymerized with 0.2% (*w*/*v*) human fibrin (**A**) or 0.14% human fibrinogen (**B**). Plasmin (2 μg) was used as positive control. Led: *Lentinula edodes*, Pos: *Pleurotus ostreatus*, Per: *Pleurotus eryngii* and Aae: *Agrocybe aegerita*.

Analysis by mono-dimensional zymography (Figure 5) was performed to determine the composition and the molecular mass of the fibrinolytic (gel A) and fibrinogenolytic (gel B) enzymes present in the crude extracts. Human plasmin was used as a positive control. In both zymograms (gels A and B), all samples showed a proteolytic pattern characterized by the presence of well-defined digestion bands. In the extract obtained from the fruiting bodies of *L. edodes* (Led) a single fibrinolytic band of about 50 kDa was detected in both gels. This band showed a molecular weight higher than that of the fibrinolytic enzyme (LEFE) (of about 38 kDa by SDS-PAGE) purified from *Lentinus edodes* GNA01 and identified as a metalloprotease [21]. *P. ostreatus* (Pos) extract showed the presence of two different fibrinolytic bands, the first with a high molecular weight which failed to migrate into the resolving gel, and the second of about 40 kDa (gel A) and 50 kDa (gel B). Choi and Shin [18] purified a fibrinolytic enzyme from the fruiting body of Pos which displayed a different molecular weight when analyzed by gel filtration (24 kDa) and SDS-PAGE (12 kDa). The authors explained this discrepancy in the molecular weight hypothesizing that the native enzyme is a dimer. Liu et al. [37] purified from the submerged culture fermentation, a fibrinolytic enzyme with molecular mass determined by gel filtration and SDS-PAGE of 13.6 and 18.2 kDa, respectively. Regarding the extract of *P. eryngii* (Per), the fibrinolytic pattern (gel A) is characterized by the presence of four different bands between 80 kDa and 32 kDa, the highest activity was detected at 80 kDa, the other minor bands showed an apparent molecular mass of 44, 42 and 32 kDa, respectively. In gel B, only the two bands of 44 and 42 kDa were observed. In addition, a major band at the interface between the upper and lower gel was detected, probably corresponding to the aggregation of the other bands. Cha et al. [20] reported on a small fibrinolytic enzyme of 14 kDa purified from the fruiting body of *Pleurotus eryngii*, completely inhibited by PMSF. Finally, for the extract of *A. aegerita* (Aae) in gel A, four different fibrinolytic bands of 120, 60, 44 and 40 kDa were detected, whereas in the fibrinogen zymogram (gel B) only a single band of about 82 kDa was observed. Recently, a novel fibrinolytic enzyme (ACase) was isolated from fruiting bodies of *Agrocybe aegerita*. The enzyme was found to be a heterodimer with molecular mass of 31.4 and 21.2 kDa by SDS-PAGE and appeared as a single band on native-PAGE and fibrin-zymogram [13]. The discrepancy between the molecular weight of the fibrin(ogen)olytic activity observed by us in comparison with other authors might be due to the different techniques used for the detection of the enzymes or to the fact that under non-reducing conditions the enzymes might migrate as oligomeric forms. On the other hand, the analysis of the fibrinolytic activity in the presence of different proteinase inhibitors, also evidenced in our extracts the presence of metalloproteases and serine proteases detected by others [18,20,21,37].

## 3. Materials and Methods

### 3.1. Chemicals

All the reagents used were of the highest grade and were purchased from Sigma-Aldrich (St. Louis, MO, USA), Carlo Erba (Milan, Italy), Bio-Rad Laboratories (Segrate, Italy) and GE Healthcare (Uppsala, Sweden).

### 3.2. Mushroom Samples

Four species of edible mushrooms: *Lentinula edodes* (strain BIO332), *Pleurotus ostreatus* (strain BIO334), *Pleurotus eryngii* (strain BIO175) and *Agrocybe aegerita* (strain BIO262) were provided by the Bioagritest Mycological Research Center (Pignola, Italy). The fungal species were preliminarily identified morphologically and genetically through optical microscopic observations of the fungal structures and molecular analysis of DNA. The strains of the different species used are kept in the Bioagritest mycoteca. Pure cultures of the different strains were prepared on acidified potato dextrose agar (PDA) with antibiotic additives (PDA 39 g/L, lactic acid 1.25 mL/L, streptomycin sulphate 0.2 g/L). The inoculated Petri dishes were incubated at 25 ± 2 °C in the dark for ten days, then used for the inoculation of growing medium after the completion of mycelium. Medium for cultures of *P. eryngii*, *P. ostreatus* and *A. aegerita* was prepared by mixing 16.25% cellulosic matrix (spelled chaff, corn chips, thistle chips and wheat sawdust at the ratio of 1:1:1:1), 3.75% calcium carbonate, 15% exhausted sugar beet pulp and 65% water, whereas, for *L. edodes*, the cellulosic matrix was composed only of oak sawdust. One polypropylene bottle (for *P. eryngii*, *P. ostreatus* and *A. aegerita*) or packets (for *L. edodes*) were filled with the respective medium for culture, sterilized (in autoclave for 1 h at 121 °C), inoculated with mycelium (23 ± 2 °C for 15–30 days) and used for spawning. After completion of mycelial growth, bottles/packets were uncapped/cut and stored in a conditioned chamber (15–23 °C, 70–85% relative humidity and 180–250 lux). Carbon dioxide concentration was monitored and controlled instrumentally. One flush of mushrooms in each bottle or bag was harvested. Fruiting bodies were cleaned, cut into pieces, and dried at 37 °C in a hot-air drier, then ground to a powder and stored in a sealed polyethylene bag at room temperature. 

### 3.3. Preparation of Crude Extracts

For each species, 1 g of powder was cold homogenized with 40 mM Tris HCl, pH 7.0 (1:10, *w*:*v*). Samples were stirred on ice for 4 h and centrifuged (10,000× *g*, 4 min at 20 °C). The supernatants, corresponding to the crude extracts, were filtered on Whatman 3 paper discs, then aliquoted and immediately used for analysis or stored at −20 °C.

### 3.4. Protein Estimation and Assessment of Total Proteolytic Activity

Protein concentration was determined according to the method of Bradford [38] using a calibration curve obtained with bovine serum albumin (BSA) as standard protein. Readings were made at 595 nm using the UltraSpec 2000 spectrophotometer (Amersharm Pharmacia Biotech, UK). Results were expressed in terms of mg of protein/g of dry weight. Total proteolytic activity was assessed by using the spectrophotometric method based on azocasein [39]. Briefly, 0.1 mL of crude extracts were added to 0.4 mL of 1% azocasein (Sigma-Aldrich, St. Louis, MO, USA), in 40 mM Tris-HCl buffer pH 7.0 at 37 °C. The reaction was stopped after 90 min by the addition of 0.5 mL 10% trichloroacetic acid (TCA) and centrifuged for 6 min at 8000 rpm (Amicon microcentrifuge MC-13; Amicon, Beverly, MA, USA). To the supernatants, separated from the undigested substrate, an equal volume of 0.5 N NaOH was added, then the absorbance at 440 nm of the released dye was recorded. One unit of total proteolytic activity (U) was defined as the amount of enzyme yielding 0.001 unit of absorbance per min at 440 nm under the assay conditions. The assay included an appropriate blank, in which TCA was added before the substrate. Total proteolytic activity was reported as specific activity: U/mg of protein.

### 3.5. Fibrin Plate Assay

Fibrinolytic activity was determined by using the method of Astrup and Mullertz [40] with some modification. Fibrin agarose plates (1 mm thickness) were prepared as follows: 5 mL of 0.8% human fibrinogen solution in 40 mM Tris-HCl buffer, pH 6.8 was mixed with the same volume of 2% agarose solution along with 1 mL of thrombin solution (200 U/mL) (Sigma-Aldrich, St. Louis, MO, USA) and poured into a Petri dish. The plates were allowed to stand for 1 h at room temperature to allow formation of a fibrin clot layer. Then, 20 μL of crude extracts were carefully placed onto the plates and incubated for 4 h at 37 °C. Human plasmin was used as a positive control. Fibrinolytic activity of samples, visible as clear lysis circles, was quantified by using a calibration curve obtained by the relation between the lytic area and the corresponding amount of human plasmin (Sigma-Aldrich, St. Louis, MO, USA) loaded on the plate, used as standard enzyme. Fibrinolytic activity was expressed as µg of plasmin equivalent (PE).

### 3.6. Spectrophotometric Detection of Fibrin(ogen)olytic Activity

The activity of the fibrin(ogen)olytic enzymes were measured spectrophotometrically, using the chromogenic substrates tosyl-Gly-Pro-Lys-p-nitroanilide (Sigma-Aldrich, St. Louis, MO, USA) specific for plasmin. The substrate (1.5 mM) was prepared in a solution of 50% ethanol in 20 mM sodium phosphate pH 6.8. For the assay, 50 µL of crude extract was mixed with 100 µL of substrate and 650 µL of sodium phosphate 20 mM pH 6.8. The mixture was incubated at 37 °C for 30 min and the absorbance at 405 nm of the released p-nitroanilide was measured (UltraSpec 2000 spectrophotometer, Amersharm Pharmacia Biotech). Enzyme activity was calculated using a calibration curve obtained using human plasmin (Sigma-Aldrich, St. Louis, MO, USA) as standard enzyme, and expressed as µg of plasmin equivalents (PE)/mg of protein.

#### 3.6.1. Effect of pH and Temperature

The influence of pH on the fibrin(ogen)olytic activity was evaluated within a pH range of 3–10 at 37 °C. The buffers used and their pH ranges were: 40 mM glycine-HCl (pH 3.0), 40 mM sodium acetate buffer (pH 4.0–6.0), 40 mM Tris-HCl (pH 7.0–9.0) and 40 mM carbonate buffer (pH 10.0). The maximum activity was considered as 100%, while the activity at various pHs were expressed as a relative percentage compared to 100%. The optimal temperature for enzymatic activity was determined at various temperatures (20–60) °C. The maximum activity was considered as 100%, while the activity at various temperatures were expressed as a relative percentage compared to 100%.

#### 3.6.2. Effect of Protease Inhibitors

To determine the effects of specific proteinases inhibitors (Sigma-Aldrich, St. Louis, MO, USA) on the fibrin(ogen)olytic enzymes, the crude extracts were incubated in the presence of different enzyme inhibitors such as iodoacetamide (30 mM) for cysteine proteinases; pepstatin A (2 μM) for aspartic proteinases; phenylmethylsulphonyl fluoride (PMSF) (4 mM) for serine proteinases and 1,10-phenanthroline (PA) (20 mM) for metalloproteinases. Aliquots of each inhibitor stock solution were mixed separately with crude extracts, incubated for 1 h at 25 °C and then added to the synthetic substrate tosyl-Gly-Pro-Arg-p-nitroanilide as described before. Residual activity was calculated in relation to a negative control represented by the extract incubated without inhibitors.

### 3.7. Analysis of the Degradation Pattern of Fibrinogen and Fibrin by SDS-PAGE

A total of 1 mL of 0.4% human fibrinogen or 0.2% human fibrin (Sigma-Aldrich, St. Louis, MO, USA) in 20 mM Tris-HCl, pH 6.8 was incubated separately with 0.1 mL of each crude extract (1 μg/μL of protein) at 37 °C. At various time intervals, aliquots of 7.5 μL of the fibrinogen reaction mixture or 12.5 μL for fibrin reaction mixture were taken and mixed with 12.5 μL of denaturing loading buffer (20% glycerol, 8% SDS and 2% β-mercaptoethanol) and incubated for 5 min at 100 °C. The degradation patterns of fibrin(ogen)olysis were analyzed by 10% SDS-polyacrylamide gel according to the method of Laemmli [41]. The data have been shown as supplementary material (Figure S1).

### 3.8. Zymographic Analysis of Fibrinolytic and Fibrinogenolytic Activities

Fibrinolytic and fibrinogenolytic activities were also determined by zymography. Aliquots of crude extracts containing 8 μg of proteins were supplemented with 20 μL of electrophoresis non-reducing loading buffer: 4% (*w*/*v*) SDS, 12% (*w*/*v*) glycerol, 0.01% (*w*/*v*) bromophenol blue, 50 mM Tris–HCl (pH 6.8). Two μg of human plasmin supplemented with 20 μL of loading buffer was used as a positive control. Samples were then separated under non-reducing conditions in a 10% (*w*/*v*) polyacrylamide gel copolymerized with 0.14% (*w*/*v*) human fibrinogen or 0.2% (*w*/*v*) human fibrin. Stacking gels contained 4% (*w*/*v*) polyacrylamide. Electrophoresis was carried out at 4 °C for 80 min at 150 V constant using a Bio-Rad Miniprotean apparatus (Bio-Rad Laboratories). After electrophoresis, the gels were washed (2 × 20 min) in 2.5% (*w*/*v*) Triton X-100, 20 mM Tris-HCl buffer (pH 6.8) (washing buffer) in order to remove SDS, then incubated for 16 h at 37 °C in 20 mM Tris-HCl, pH 6.8. At the end of the incubation the gels were stained with Coomassie Brilliant Blue. After destaining of the gel, the fibrinolytic and fibrinogenolytic activities were detected as with bands of digestion on the blue background of the gel.

## 4. Conclusions

In this study different techniques have been used to assess and compare the fibrin(ogen)olytic activity present in the crude extracts obtained from the fruiting bodies of the cultivated edible mushrooms *L. edodes*, *P. ostreatus*, *P. eryngii* and *A. aegerita*. All the studied mushrooms displayed fibrin(ogen)olytic activity with an optimal pH and temperature close to the physiological values of humans. Based on these results, the crude extracts or the powdered mushrooms can be used as functional ingredients for the development of new functional food that may act as thrombolytic agents. 

## Figures and Tables

**Table 1:** Protein content, total proteolytic and fibrin(ogen)olytic activity of crude extracts from the fruiting bodies.

	Proteins	Total Proteolytic Activity	Fibrin(ogen)olytic Activity	Specific Fibrin(ogen)olytic Index
Samples	(mg/g DW)	Specific Activity (U/mg prot)	Specific Activity (µg PE/mg prot)	SFI: µg PE/U
Led	7.67 ± 0.25 ^a^	85.03 ± 2.32 ^a^	23.25 ± 0.16 ^a^	0.27 ± 0.01 ^a^
Pos	8.32 ± 0.15 ^b^	107.65 ± 6.24 ^b^	39.14 ± 1.01 ^b^	0.36 ± 0.02 ^b^
Per	9.02 ± 0.20 ^c^	92.89 ± 3.26 ^c^	35.09 ± 0.40 ^c^	0.38 ± 0.02 ^b^
Aae	9.81 ± 0.39 ^d^	84.69 ± 1.91 ^a^	22.11 ± 0.13 ^d^	0.26 ± 0.03 ^a^

Led: *Lentinula edodes*; Pos: *Pleurotus ostreatus*; Per: *Pleurotus eryngii*; Aae: *Agrocybe aegerita*. Total proteolytic activity was carried out using azocasein as substrate and expressed as U/mg prot, were U corresponds to the amount of enzyme yielding 0.001 unit of absorbance at 440 nm per min. Fribin(ogen)olytic activity was carried out using tosil-Gly-Pro-Lys-p-nitroanilide as substrate and expressed as µg Plasmin Equivalent (PE)/mg prot. Specific Fribin(ogen)olytic Index (SFI): corresponds to the ratio between amidolytic activity and total proteolytic activity. Values are reported as mean ± s.e.m. of two independent experiments performed in triplicate (n = 6). The mean values with different letters (in the same column) are significantly different as analyzed by ANOVA (*p* < 0.05, Tukey’s test).

**Table 2:** Fibrinolytic activity of crude extracts assessed by fibrin plate.

Samples	Area of Lytic Zone (cm^2^)	Diameter (cm)	Plasmin Equivalent (PE) (μg)
*Lentinula edodes*	4.15 ± 0.18 ^a^	2.30 ± 0.10 ^a^	2.89 ± 0.12 ^a^
*Pleurotus ostreatus*	6.15 ± 0.28 ^b^	2.80 ± 0.13 ^b^	4.25 ± 0.19 ^b^
*Pleurotus eryngii*	4.91 ± 0.21 ^c^	2.50 ± 0.11 ^c^	3.41 ± 0.15 ^c^
*Agrocybe aegerita*	3.46 ± 0.19 ^d^	2.10 ± 0.07 ^d^	2.42 ± 0.13 ^d^

Values are reported as mean ± s.e.m. of two independent experiments performed in duplicate (n = 4). The mean values with different letters (in the same column) are significantly different as analyzed by ANOVA (*p* < 0.05, Tukey’s test).

**Table 3:** Effect of protease inhibitors on fibrin(ogen)olytic enzymes of crude extracts.

	Residual Enzymatic Activity (%)			
Inhibitor	*Lentinula edodes*	*Pleurotus ostreatus*	*Pleurotus eryngii*	*Agrocybe aegerita*
Control	100 ^a^	100 ^a^	100 ^a^	100 ^a^
PMSF	100 ^a^	79.8 ± 4.4 ^b^	54.1 ± 3.3 ^b^	29.9 ± 1.8 ^b^
PA	4.5 ± 0.2 ^b^	25.0 ± 3.8 ^c^	49.1 ± 5.2 ^a^	79.6 ± 5.3 ^c^
IAA	100 ^a^	100 ^a^	100 ^a^	100 ^a^
PEP	100 ^a^	100 ^a^	100 ^a^	100 ^a^

Activity of the fibrin(ogen)olytic enzymes was carried out using tosil-Gly-Pro-Lys-p-nitroanilide as substrate. Control: was the activity (100%) determined in the absence of inhibitors; PMSF: Phenylmethylsulfonyl fluoride; PA: 1, 10 Phenantroline; IAA: Iodoacetamide; PEP: Pepstatin. Values are reported as mean ± s.e.m. of two independent experiments performed in triplicate (n = 6). The mean values with different letters (in the same column) are significantly different as analyzed by ANOVA (*p* < 0.05, Tukey’s test).

## Data Availability

Not applicable.

## Supplementary Materials

The following supporting information can be downloaded at: https://www.mdpi.com/article/10.3390/molecules27238145/s1, Figure S1: Analysis of fibrin(ogen)olysis patterns by SDS-PAGE.

## Sample Availability

Samples of the compounds are not available from the authors.
