# Supplementary material for: Edible Mushrooms as Source of Fibrin(ogen)olytic Enzymes: Comparison between Four Cultivated Species
Source: Molecules. 2022 Nov 23;27(23):8145. doi: 10.3390/molecules27238145 (PMC9738689; doi:10.3390/molecules27238145)
Supplement: Supplementary file 1 [file molecules-27-08145-s001.zip › molecules-2036899-supplementary.pdf]

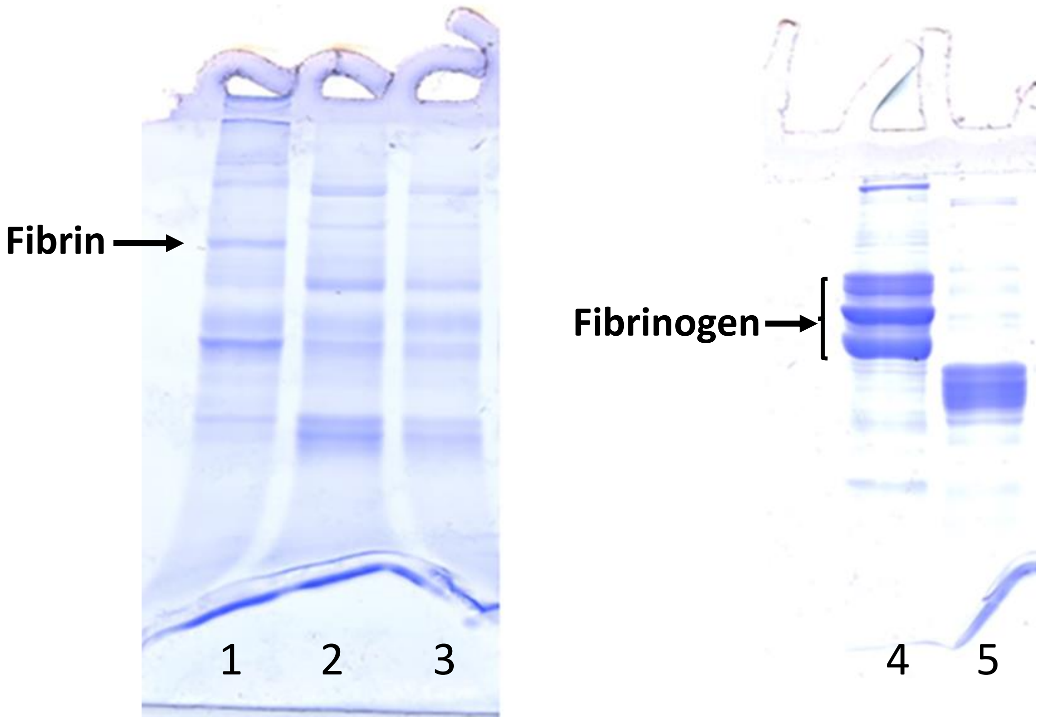

Figure S1. Analysis of fibrin(ogen)olysis patterns by SDS-PAGE. Fibrinogenolysis (right panel) and fibrinolysis (left panel) pattern exhibited by human plasmin (positivfe control). 0,8 µg of human plasmin were incubated at 37 °C with 0.4% human fibrinogen or 0.2 % human fibrin in 20 mM Tris-HCl, pH 6.8, then aliquots of the reaction mixture were removed at different time intervals and analyzed by SDS-PAGE.

Lane 1: human fibrin in 20 mM Tris-HCl, pH 6.8; Lane 2: human fibrin incubated with human plasmin for 20 min; Lane 3: human fibrin incubated with human plasmin for 40 min; Lane 4: human fibrinogen in 20 mM Tris-HCl, pH 6.8; Lane 5: human fibrin incubated with human plasmin for 5 min.
